# Surgical Management of Locally Advanced and Metastatic Gallbladder Cancer

**DOI:** 10.3390/cancers17243952

**Published:** 2025-12-11

**Authors:** Mitchell Breitenbach, Paul Burchard, Veer Kothari, Darren Carpizo

**Affiliations:** 1 Department of Surgery, Division of General Surgery, University of Rochester Medical Center, Rochester, NY 14642, USA; paul_burchard@urmc.rochester.edu; 2 Department of Surgery, Division of Surgical Oncology, University of Rochester Medical Center, Rochester, NY 14642, USA; 3 Wilmot Cancer Institute, University of Rochester Medical Center, Rochester, NY 14642, USA

**Keywords:** gallbladder cancer, locally advanced disease, metastatic disease, surgical resection, radical cholecystectomy, hepatic resection, bile duct resection, metastasectomy, portal vein embolization, systemic therapy

## Abstract

Advances in systemic therapy for gastrointestinal malignancies have opened the door for surgical resection of tumors previously deemed unresectable. Of these tumors, gallbladder cancer has a particularly poor prognosis due to the aggressive nature of the disease. The preferred systemic therapy regimen for gallbladder cancer has progressed from single-agent chemotherapy to multi-agent therapy including immune checkpoint inhibitors, with additional targeted therapies currently under investigation. These advancements have provided patients with historically unresectable tumors with a bridge to surgical resection and a hope of extended survival or cure. Surgical options for locally advanced tumors have expanded, and experienced centers perform a variety of operations to achieve resection with negative margins, including extended liver resection, bile duct resection, vascular reconstruction, and adjacent organ resection. There is also growing evidence that patients with Stage IV disease may benefit from resection of their primary tumors and metastases. This review outlines the current treatment practices for patients with locally invasive and metastatic gallbladder cancer as well as emerging treatment options.

## Simple Summary

Surgical resection with negative margins is the only curative treatment for gallbladder cancer. Unfortunately, many patients with this uncommon malignancy are diagnosed at a late stage with tumors that have either invaded their surroundings or have metastasized. Surgical management for these tumors has thus historically been limited. Throughout the 21st century, the systemic therapy algorithm for gallbladder cancer has progressed, and extended resection options have been explored, leading to an increased focus on achieving margin-negative resection and cure. This review provides an overview of the role of surgery for locally advanced and metastatic gallbladder cancer, looking at the range of surgical options for locally invasive tumors and the potential benefits of surgical resection for metastatic disease.

## 1. Introduction

Surgical resection with negative margins provides patients the best chance of cure for gastrointestinal solid organ malignancies. Early-stage cancers tend to be most amenable to this treatment, while the role of surgery in locally advanced or metastatic tumors has historically been limited. However, advances in systemic therapy have allowed for increased ability to resect previously unresectable tumors. For several gastrointestinal malignancies, including colorectal and pancreatic cancer, resection of the primary tumor with or without metastasectomy has been shown to provide a survival benefit in metastatic disease [1,2]. This review aims to explore the surgical management of locally advanced (T3 or T4) gallbladder tumors and to provide insight into the role of surgery for metastatic gallbladder cancer (GBC).

An estimated 122,462 cases of GBC were diagnosed in 2022, with 89,031 mortalities worldwide [3]. Although GBC accounts for less than 1% of all cancers, its incidence and mortality have greatly increased in the past several decades and are both projected to continue rising [4,5]. While most cases arise in Asian countries, gallbladder and biliary cancers led to an estimated 4400 deaths in the United States in 2025 [6]. Major risk factors for the development of GBC include female sex, Asian or Latin American ethnicity, obesity, and a history of gallstones or cholecystitis [4,5,7]. Of those diagnosed with GBC, there is an increased risk of death in patients of advanced age and black race [8].

The predominant histology of GBC is adenocarcinoma, accounting for about 9 in 10 cases, with papillary, squamous, and adenosquamous tumors seen less commonly [9,10]. Two major pathways to the development of GBC have been proposed [11]. The metaplasia-dysplasia pathway, which is believed to account for most GBCs, involves long-lasting inflammation from gallstones or chronic cholecystitis driving metaplasia, which progresses to dysplasia, carcinoma in situ, and ultimately adenocarcinoma [12,13]. Alternatively, a minority of GBCs are thought to develop from an adenoma-carcinoma sequence with damage of biliary tract epithelium from anomalous pancreatic enzyme reflux leading to hyperplasia, dysplasia, and carcinogenesis [14].

While many GBCs can be diagnosed prior to any interventions, nearly half are discovered incidentally on pathology following laparoscopic cholecystectomy [10]. Regardless of how they are detected, diagnostic workup includes cross-sectional imaging of the chest, abdomen, and pelvis. Laboratory testing includes liver function tests and the tumor markers CA 19-9 and CEA, while genetic testing may also be performed to assess for inherited cancer syndromes [15]. Tissue diagnosis is not required before surgical treatment, but staging laparoscopy is recommended as it can detect disseminated disease or identify tumors as unresectable [16,17,18]. For those who present with jaundice and undergo surgical resection, cholangiography should be utilized to rule out biliary obstruction. A tissue sample with mutational analysis may be especially beneficial in guiding therapy for unresectable tumors, as one review of resected GBCs found half of the analyzed tumors to exhibit at least one mutation with an actionable therapeutic target [19].

The American Joint Committee on Cancer (AJCC) 8th edition GBC staging system classifies tumors based on their depth of invasion (T stage), lymph node involvement (N stage), and presence of distant metastasis (M stage) [20]. Choice of surgical resection is guided by the AJCC T stage. For tumors that invade only the lamina propria (T1a), simple cholecystectomy is adequate. For any tumor that invades further (T1b and above), radical cholecystectomy is recommended, including en bloc hepatic resection of segment IVb and V and portal hepatis lymphadenectomy in addition to gallbladder removal [15].

Surgical resection with negative margins is the only potential cure for patients with GBC. Unfortunately, radical cholecystectomy alone may not adequately control locally advanced disease. In the United States, 39.9–43.7% of patients with GBC will present with tumors that invade through the serosa or into adjacent organs and structures, and 17.6–18.3% will present with metastatic disease [21,22]. For these patients, a combination of systemic therapy and more extensive resection may be necessary to achieve R0 status. Studies analyzing operative intervention for locally advanced and metastatic GBC are often of limited sample size due to the relative rarity of this cancer and the infrequency of these operations; this review aims to summarize the existing data to provide a treatment framework for these complex malignancies.

## 2. Advances in Systemic Therapy for Advanced Biliary Cancers

The landscape of systemic therapy for locally advanced and metastatic GBC has evolved throughout the 21st century. Initial chemotherapy regimens for patients whose cancers were deemed unresectable most commonly included gemcitabine or fluoropyrimidines and were based on extrapolation of data from other cancers, case reports, or small trials [23,24]. Although frequently utilized, gemcitabine monotherapy demonstrated variable response rates and overall survival [25].

In the mid-2000s, the ABC-02 trial was conducted across 37 centers in the United Kingdom. This phase 3 trial compared gemcitabine monotherapy to combination therapy with gemcitabine and cisplatin for patients with unresectable, recurrent, or metastatic biliary tract cancers. The authors found that patients treated with gemcitabine and cisplatin had improved median tumor response (81.4% vs. 71.8%), progression-free survival (8.0 vs. 5.0 months), and overall survival (11.7 vs. 8.1 months) compared to gemcitabine alone (Table 1) [26]. The SWOG S1815 trial later looked at the addition of nab-paclitaxel to gemcitabine and cisplatin for this patient population but failed to find a significant difference in overall or progression-free survival compared to gemcitabine and cisplatin [27].

For the following decade after ABC-02, gemcitabine and cisplatin remained the standard of care for this patient population until the investigation of immune checkpoint inhibitor use in the TOPAZ-1 and KEYNOTE-966 trials. From 2019 to 2020, the TOPAZ-1 trial enrolled patients with unresectable, locally advanced, or metastatic biliary tract adenocarcinomas from more than 100 sites worldwide. Patients were randomized to receive gemcitabine and cisplatin combined with either durvalumab (a PD-L1 inhibitor) or placebo. The group that received durvalumab had improved median overall survival (12.9 vs. 11.3 months) and also contained a higher proportion of patients surviving at least 2 years following randomization (23.6% vs. 11.5%), supporting the addition of immune checkpoint inhibitors to the standard chemotherapy regimen [28,29]. Use of pembrolizumab, another immune checkpoint inhibitor, was subsequently supported by the KEYNOTE-966 trial, in which patients assigned to gemcitabine, cisplatin, and pembrolizumab had significantly increased median overall survival compared to gemcitabine and cisplatin alone (12.7 vs. 10.9 months) [30].

Based on these studies, current guidelines suggest that neoadjuvant systemic therapy be considered for patients with locoregionally advanced GBC. For unresectable or metastatic biliary tract cancers, the preferred first-line regimen is gemcitabine and cisplatin with either durvalumab or pembrolizumab. The ABC-06 trial established FOLFOX as the preferred therapy for patients who progress after first-line treatment [31]. It should be noted that neoadjuvant therapy is not recommended for patients with early-stage GBC. There is emerging evidence that chemoradiation also has a role in neoadjuvant therapy for locally advanced GBC with the POLCAGB trial, a phase III trial comparing neoadjuvant dual agent chemotherapy to chemoradiotherapy. An analysis of 124 patients enrolled from 2016–2024 showed improved overall survival in the neoadjuvant chemoradiotherapy group compared to neoadjuvant gemcitabine and cisplatin (21.8 vs. 10.1 months) [32]. While this trial demonstrates superiority of chemoradiation over dual agent chemotherapy, it is not clear how this compares to dual agent chemotherapy with immunotherapy, and future studies are necessary to determine this.

**Table 1:** Completed phase III clinical trials studying systemic therapy for biliary tract cancers.

Trial *Enrollment Years*	Patient Population	Treatment Group (n)	Control Group (n)	Outcomes
ABC-02 [26] *2002–2008*	Unresectable, recurrent, or metastatic biliary tract cancers	Gemcitabine + cisplatin (204)	Gemcitabine (206)	Treatment group with improved median OS (11.7 vs. 8.1 months) and PFS (8.0 vs. 5.0 months)
SWOG S1815 [27] *2018–2021*	Unresectable, locally advanced, or metastatic biliary tract cancers	Gemcitabine + cisplatin + nab-paclitaxel (294)	Gemcitabine + cisplatin (147)	No difference in OS or PFS between groups
POLCAGB [32] *2016–2024*	Unresectable locally advanced gallbladder cancers	Gemcitabine + radiotherapy followed by gemcitabine + cisplatin (60)	Gemcitabine + cisplatin (64)	Treatment group with improved median OS (21.8 vs. 10.1 months) and R0 resection rate (51.6% vs. 29.7%)
TOPAZ-1 [28] *2019–2020*	Unresectable, locally advanced, or metastatic biliary tract cancers	Gemcitabine + cisplatin + durvalumab (341)	Gemcitabine + cisplatin + placebo (344)	Treatment group with improved median OS (12.9 vs. 11.3 months) and PFS (7.2 vs. 5.7 months)
KEYNOTE-966 [30] *2019–2021*	Unresectable, locally advanced, or metastatic biliary tract cancers	Gemcitabine + cisplatin + pembrolizumab (533)	Gemcitabine + cisplatin + placebo (536)	Treatment group with improved median OS (12.7 vs. 10.9 months)
ABC-06 [31] *2014–2018*	Locally advanced or metastatic biliary tract cancers that progressed on gemcitabine and cisplatin therapy	Active symptom control + FOLFOX (oxaliplatin + L-folinic acid + fluorouracil) (81)	Active symptom control alone (81)	Treatment group with improved median OS (6.2 vs. 5.3 months)
BILCAP [33] *2006–2014*	Resected biliary tract cancers	Adjuvant capecitabine (210)	Observation alone (220)	Treatment group with improved median OS (53 vs. 36 months) and RFS (25.9 vs. 17.4 months) in per-protocol analysis

If patients ultimately undergo resection of their GBC, adjuvant chemotherapy with capecitabine is preferred based on the BILCAP trial, which showed improved median recurrence-free survival (25.9 vs. 17.4 months) and overall survival (53 vs. 36 months) in the per-protocol group of patients treated with adjuvant capecitabine [33]. Other acceptable adjuvant treatments include gemcitabine monotherapy, gemcitabine combination therapy, or 5-fluorouracil monotherapy [15], while the ACCORD trial recently suggested a benefit from adjuvant immune checkpoint inhibitor therapy and chemoradiation as well [34]. Adjuvant therapy is most likely to benefit high-risk patients, such as those with node-positive disease or patients with positive margins [35].

## 3. Surgical Management of Locally Advanced Disease

R0 resection is the only established curative treatment for GBC. For gallbladder carcinoma in situ or tumors confined to the mucosa, a simple cholecystectomy is a sufficient operation with 5-year overall survival rates of nearly 100% [36]. For deeper tumors, the resection of choice becomes radical cholecystectomy with en bloc liver resection of segments IVb and V, as well as lymphadenectomy of the porta hepatis, gastroduodenal ligament, and retroduodenal nodes. Patients with T2 or larger tumors have demonstrated improved survival after radical cholecystectomy compared to simple cholecystectomy; these survival differences are less established for T1b tumors, but radical cholecystectomy remains the operation of choice [37,38].

Unresectable GBC has traditionally been defined by gross metastases (including lymph node metastases not confined to locoregional nodes), malignant ascites, or tumor invasion that would preclude R0 resection, such as significant involvement of major vasculature or the hepatoduodenal ligament. While some locally advanced tumors meet these definitions, others maintain hope of achieving margin-negative resections. These resections may need to extend beyond the gallbladder and liver segments IVb or V, sometimes requiring extended hepatectomy, excision of the extrahepatic bile ducts, vascular resection/reconstruction, or en bloc resection of adjacent organs.

The extent of liver resection in most patients with resectable locally advanced GBC is similar to that of patients with early-stage disease and can often be limited to wedge resection or minor hepatectomy [39,40]. Anatomic resection of IVb and V has not been seen to improve survival compared to wedge resection and is associated with increased postoperative complications [41]; still, this may be necessary for locally advanced GBCs that invade the hepatic parenchyma. There is no established consensus for margin size during GBC excision, but patients with negative margins after surgery have significantly improved survival compared to those with microscopic involvement of the margin [42]. For gallbladder tumors invading further than the adjacent liver segments, IVb/V bisegmentectomy may not be sufficient to achieve an R0 resection; thus, a minority of patients will require removal of 3 or more liver segments. Major hepatectomy is associated with a significant risk of morbidity and mortality following resection but does not provide a benefit in survival [43,44]. Thus, if not required due to the extent of tumor invasion, empiric major hepatectomy should not be performed.

If a major hepatectomy is required for GBC, one must determine how much liver will remain following resection. To avoid post-hepatectomy liver failure, a minimum of 25% future liver remnant (FLR) is recommended for patients with healthy hepatic parenchyma [45]. Patients with GBC are at higher risk for post-hepatectomy liver failure, and thus a higher threshold of 30–40% FLR has been proposed [46,47]. Preoperative evaluation of estimated postoperative liver remnant size and function is therefore of paramount importance. Measuring liver volume alone does not account for the function of a patient’s liver; rather, it is recommended to perform [99mTc] Tc-mebrofenin hepatobiliary scintigraphy, which assesses for liver function of the FLR [48]. This imaging modality has been demonstrated to be a useful preoperative diagnostic tool to guide the treatment of patients with suboptimal FLR [49].

In the case of inadequate predicted FLR, portal vein embolization (PVE) can be utilized as a preventative measure against post-hepatectomy liver failure. By eliminating portal blood flow to the segment of liver being resected, this procedure allows the post-resection liver remnant to hypertrophy, reducing the risk of hepatic failure postoperatively. Volume of the non-embolized segments can increase by 9.7–11.9% with morbidity of this procedure being rare and frequently minor when it occurs [50]. PVE is an established method in GBC for patients requiring major hepatectomy with inadequate FLR [47,51]. Other strategies to increase FLR include liver venous deprivation (LVD) and associated liver partition and portal vein ligation for staged hepatectomy (ALPPS). LVD entails embolizing not only the portal vein branch supplying the portion of liver to be resected, but also the corresponding hepatic vein branch. This has been shown to increase FLR to a greater extent than PVE alone. ALPPS involves a first-stage procedure where tumors in the FLR are resected, the portal vein branch feeding the expected portion of liver to be resected is ligated, and the FLR is divided from the portion of liver to be resected. ALPPS thus has the potential to shorten the interval between the FLR augmentation procedure and hepatectomy [52,53].

Resection of the common hepatic or common bile duct is not empirically recommended for GBC; if uninvolved by tumor, bile duct resection does not provide a survival advantage, and there is a significantly higher rate of postoperative complications in patients undergoing this procedure [43,54]. However, extrahepatic bile duct excision may be necessary if there is direct or microscopic tumor involvement of these structures, as can be seen with some locally advanced GBCs [16]. One review from a high-volume center showed a survival advantage of tumor resection that included extrahepatic bile duct resection over patients who did not have their disease resected, suggesting that this procedure may be beneficial if performed in a center with the appropriate expertise [55].

Locally advanced GBCs may also necessitate vascular resection or the resection of adjacent organs aside from the liver and bile ducts in the case of tumor involvement. The portal vein, proper hepatic artery, and hepatic artery branches are in close proximity to the gallbladder and can be involved. While reconstruction of these vessels is possible, these procedures risk significant morbidity and mortality [56]. Commonly involved organs include the colon, pancreas, duodenum, and stomach. As with extended liver resection and bile duct resection, adjacent organ resection has not been shown to improve long-term survival and can lead to additional morbidity depending on the resected organ [43]. Pancreaticoduodenectomy specifically poses a significantly increased risk of postoperative complications and mortality [57,58]. For all extended resections for GBC, the hope for R0 resection must be weighed against the risk of surgical morbidity and mortality when selecting and counseling patients.

## 4. The Role of Surgery in Metastatic Disease

Historically, treatment recommendations for metastatic GBC, in line with other solid organ malignancies, have not included surgery. Rather, management has focused on systemic therapy, clinical trials, and supportive care. With these treatments, patients with GBC have a dismal prognosis with a 5-year overall survival of less than 5% [59]. The survival benefit of primary tumor removal in other metastatic gastrointestinal malignancies raises the question of whether these trends also apply to metastatic GBC.

Several studies have shown that patients with metastatic GBC who underwent resection of their primary tumor had significantly higher overall survival compared to matched patients who did not undergo resection [60,61]. Furthermore, a retrospective review in 2023 showed a survival benefit in patients who had “low volume metastases” resected at the time of radical cholecystectomy compared to patients who underwent palliative therapy without resection. These included discrete liver metastases near the gallbladder fossa, limited peritoneal disease, or N2 lymph nodes, all of which may be resected without significantly increasing surgical morbidity [62].

**Figure 1:**
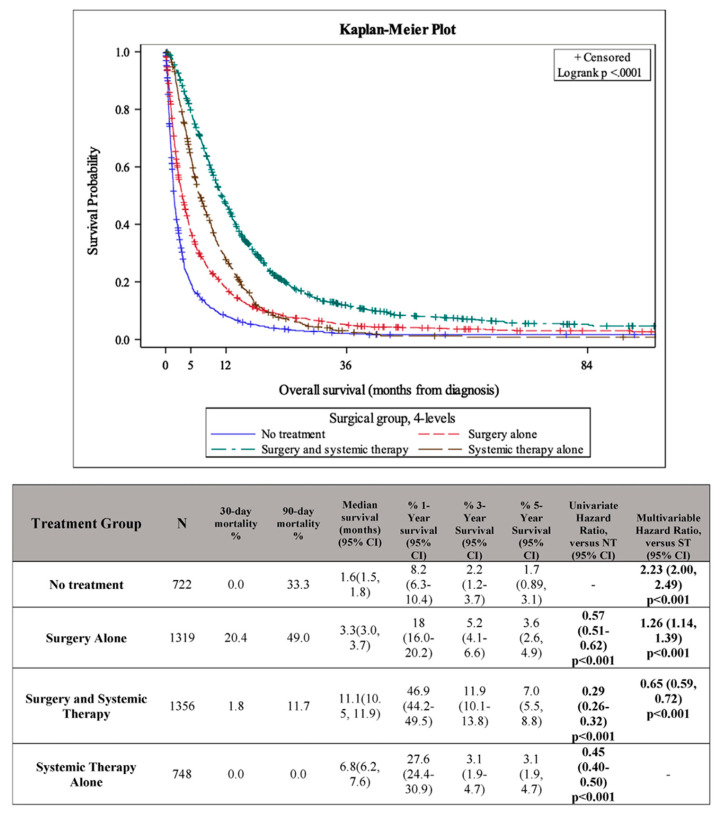
Kaplan–Meier survival curve showing overall survival of patients with stage IV GBC who underwent no treatment (NT), surgery alone, surgery and systemic therapy, or systemic therapy alone (ST). Patients with surgery in addition to systemic therapy had significantly improved survival compared to systemic therapy alone. Reproduced with permission from Darren Carpizo, “Surgery in Combination with Systemic Chemotherapy Is Associated with Improved Survival in Stage IV Gallbladder Cancer” [63]; published by Elsevier, 2022.

In 2022, our group conducted a retrospective review of the National Cancer Database that looked at the outcomes of patients with stage IV GBC who underwent surgery or systemic therapy to treat their cancer. This review of over 4000 patients found an associated benefit of surgical resection combined with systemic therapy compared to systemic therapy alone (median overall survival 11.1 vs. 6.8 months, Figure 1). In a subgroup analysis, the greatest improvement in survival was observed in those who underwent surgery in combination with multi-agent chemotherapy [63]. While this retrospective review is limited by selection bias, it suggests that primary tumor resection may be beneficial in select patient populations with metastatic GBC. As this review was published prior to the results of the TOPAZ-1 and KEYNOTE-966 trials, it remains to be seen what effect the addition of immune checkpoint inhibitor therapy will have on these trends.

## 5. Conclusions and Future Directions

Patients with locally advanced GBC often present with proximal biliary obstruction and can be difficult to distinguish from hilar cholangiocarcinomas. Surgical resection in these patients, if possible, still represents the only treatment option with the potential for long-term survival, so determination of resectability is paramount. This involves evaluation for technical resectability, accurate staging with either multi-phasic CT or MRI, and PET to evaluate for portal and celiac lymph node involvement. For patients with unresectable disease either because of technical reasons related to liver involvement or due to a more advanced stage (i.e., celiac lymph node involvement), our center initiates multi-agent chemotherapy with or without immune checkpoint inhibition, as is established as the best regimen for advanced patients. This most commonly consists of gemcitabine and cisplatin with durvalumab; however, the optimal neoadjuvant treatment regimen is unclear.

Of great importance to patients with unresectable tumors is whether neoadjuvant systemic therapy can shrink their tumors to the point where R0 resection is attainable. Ideally, the progression of systemic therapy to its current state will have concurrently improved resection rates, but robust data on this is limited. One single-center study published a conversion from unresectable to resectable in 4 of 7 patients who underwent gemcitabine monotherapy [64]. Several studies have looked at the ability of multi-agent chemotherapy before widespread use of immune checkpoint inhibitors to convert unresectable GBC to resectable disease with success rates as low as 13.5% to as high as 50.4% [65,66]. The POLCAGB trial also suggested that adding gemcitabine-based chemoradiotherapy to combination chemotherapy may improve the ability to achieve R0 resection, with the chemoradiotherapy group containing a higher proportion of patients undergoing R0 resection compared to dual-agent chemotherapy (51.6% vs. 29.7%) [32]. Data on multiple-agent systemic therapy, including immune checkpoint inhibitors, are also limited but more promising, with one single-center cohort reporting an 81% conversion rate (13/16 patients) [67]. Encouragingly, one patient from this cohort demonstrated a complete response following treatment, a phenomenon which has been described in other case reports as well [68,69]. For patients with celiac lymph node disease, who are considered stage IV, we treat them similarly to other stage IV patients.

**Figure 2:**
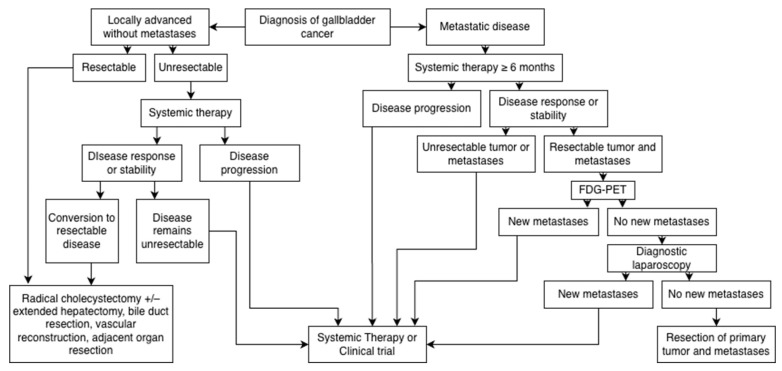
Our institution’s treatment algorithm for locally advanced and metastatic gallbladder tumors.

Our group’s previous findings challenge the notion that surgery has no role in the treatment of patients with stage IV GBC. At our institution, patients with stage IV GBC are first initiated on a systemic therapy regimen for 6–12 months. If their disease responds to this treatment, they may be considered as a candidate for resection of their primary tumor and its metastases. Those with metastasis confined to the liver are typically viewed as the best surgical candidates. The liver is the most common organ where isolated metastases are found, and patients with liver or distal lymph node metastases have been found to have improved overall and cancer-specific survival when the primary tumor is removed [70]. Once considered a candidate, patients undergo FDG-PET to assess for extrahepatic disease. In the absence of unexpected new metastases, appropriate patients are taken to the operating room and undergo diagnostic laparoscopy to assess for peritoneal metastases; if none are identified, the primary tumor and metastatic burden are resected (Figure 2).

There are multiple emerging therapies on the horizon for the treatment of GBC, specifically targeted therapies based on tumor genetics. Fibroblast growth factors (FGFs) and their receptors (FGFRs) have been implicated in the pathogenesis of GBC [71]. Thus, FGFR inhibitors are of increased interest in GBC treatment, especially considering promising results have been seen with their use in cholangiocarcinoma [72,73]. KRAS mutations are also of therapeutic interest, and KRAS inhibitors have shown efficacy in solid organ tumors with specific KRAS mutations [74]. Mismatch repair-deficient (dMMR) malignancies have recently received attention, with a phase II trial showing the potential of dostarlimab (a PD-1 inhibitor) to achieve clinical complete response of dMMR solid organ tumors [75]. This trial included patients with hepatobiliary cancers, raising the possibility of nonoperative management for a minority of GBCs that are dMMR [76].

Ongoing clinical trials studying GBC and other biliary tract cancers may continue to shed light on the utility of targeted therapy. The SAFIR-ABC10-Precision Medicine trial is a phase III umbrella trial currently enrolling patients with advanced biliary tract cancers to assess the efficacy of targeted therapy following gemcitabine, cisplatin, and durvalumab or pembrolizumab [77]. Other ongoing immunotherapy clinical trials, such as the TAPUR trial, may shed light on the potential benefits of pairing patients with advanced GBC with treatments that target their tumor’s specific mutations [78]. Table 2 shows selected ongoing clinical trials that are relevant to the treatment of GBC.

## Figures and Tables

**Table 2:** Selected ongoing clinical trials for systemic therapy in biliary tract cancers, compiled from https://clinicaltrials.gov (Accessed on 24 September 2025) [79].

Trial ID	Patient Population	Treatment	Comparator (If Applicable)	Anticipated Completion
NCT06712420 (NEOGB)	Locally advanced GBC	Neoadjuvant gemcitabine + cisplatin followed by resection	Upfront resection	2028
NCT02170090 (ACTICCA-1)	Resected biliary tract cancers	Adjuvant gemcitabine + cisplatin	Adjuvant capecitabine	2025
NCT06591520	Biliary tract cancers	Gemcitabine + cisplatin + AK112 (PD-1/VEGF inhibitor)	Gemcitabine + cisplatin + durvalumab	2027
NCT06282575	HER2-positive locally advanced or metastatic biliary tract cancers	Zanidatamab (HER2 inhibitor) + gemcitabine + cisplatin +/− durvalumab or pembrolizumab	Gemcitabine + cisplatin +/− durvalumab or pembrolizumab	2030
NCT04526106	Unresectable or metastatic solid organ tumors with FGFR2 mutations	RLY-4008 (FGFR2 inhibitor)		2027
NCT06607185	Locally advanced or metastatic solid organ tumors with KRAS mutations	LY4066434 (pan-KRAS inhibitor)		2030
NCT05615818 (SAFIR-ABC10)	Locally advanced or metastatic biliary tract cancers with targetable mutations	Gemcitabine + cisplatin +/− durvalumab or pembrolizumab, followed by therapy matched to specific mutation	Gemcitabine + cisplatin +/− durvalumab or pembrolizumab	2028

## Abbreviations

The following abbreviations are used in this manuscript:

GBC	Gallbladder cancer
AJCC	American Joint Committee on Cancer
FLR	Future liver remnant
PVE	Portal vein embolization
LVD	Liver venous deprivation
ALPPS	Associated liver partition and portal vein ligation for staged hepatectomy
FGF	Fibroblast growth factor
FGFR	Fibroblast growth factor receptor
dMMR	Mismatch repair-deficient
